# The Relationships Between the Center of Mass Position and the Trunk, Hip, and Knee Kinematics in the Sagittal Plane: A Pilot Study on Field-Based Video Analysis for Female Soccer Players

**DOI:** 10.1515/hukin-2015-0008

**Published:** 2015-04-07

**Authors:** Shogo Sasaki, Yasuharu Nagano, Satoshi Kaneko, Shoichiro Imamura, Takuma Koabayshi, Toru Fukubayashi

**Affiliations:** 1 Faculty of Health Sciences, Tokyo Ariake University of Medical and Health Sciences, Tokyo, Japan.; 2 Department of Health and Sports, Niigata University of Health and Welfare, Niigata, Japan.; 3 Graduate School of Sport Sciences, Waseda University, Saitama, Japan.; 4 Graduate School of Tokyo Ariake University of Medical and Health Sciences, Tokyo, Japan.; 5 Department of Orthopedic Surgery, Sapporo Medical University, Sapporo, Japan.; 6 Faculty of Sport Sciences, Waseda University, Saitama, Japan.

**Keywords:** two-dimensional assessment, risk factor, anterior cruciate ligament, injury prevention

## Abstract

Athletes with non-contact anterior cruciate ligament tears have common features in the sagittal plane; namely, the body’s center of mass (COM) is located posterior to the base of support, the trunk and knee joints are extended, and the hip angle is flexed. However, the relationships among these variables have not been assessed in field-based movements. This study sought to determine relationships between distances from the COM to the base of support and the trunk, hip, and knee positions in women while playing soccer. Sixty events (29 single-leg landing and 31 single-leg stopping events) were analyzed using two-dimensional video analysis. The relationships among the measurement variables were determined using the Pearson’s product-moment correlation coefficient, and stepwise multiple linear regression models were used to explore the relationships between the COM position and the kinematic variables. The distance from the COM to the base of support displayed a moderate negative relationship with the trunk angle (r = −0.623, p < .0001, r^2^ = 0.388) and a strong positive relationship with the limb angle (r = 0.869, p < .0001, r^2^ = 0.755). The limb, knee, and trunk angles were selected in the best regression model (adjusted r^2^ = 0.953, p < .0001, f^2^ = 20.277). These findings suggest that an increased trunk angle and a decreased limb angle at initial contact are associated with a safer COM position. Neuromuscular training may be useful for controlling the trunk and lower limb positions during dynamic activities.

## Introduction

Soccer is becoming popular worldwide, and the increased awareness of soccer among women is particularly interesting. According to the Fédération Internationale de Football Association (FIFA), the number of female soccer players reached 26.0 million in 2006, which is an increase of 19% compared with that in 2000, and approximately 10% of all soccer players are female (FIFA, 2007). Therefore, FIFA has considerable interest in the health and fitness of female soccer players (FIFA, 2007).

Sixty-five percent of all injuries in female soccer players playing in top-level international tournaments involve a lower extremity (Junge and Dvorak, 2007). Among these injuries, female players or staff on these teams indicate that knee injuries, particularly anterior cruciate ligament (ACL) tears, occur at a high rate, and ACL injuries require an average of 186 days of rehabilitation before resuming training and competition (Zaffagnini et al., 2014). ACL injuries in soccer are more frequent in women than in men (Arendt and Dick, 1995; Bjordal et al., 1997; Harmon and Dick, 1998; Powell and Barber-Foss, 2000). In addition, the incidence of injuries is 4–6-fold higher during games than during training sessions (Junge and Dvorak, 2004). This indicates that the mechanism of ACL injuries during games may not be replicated during training or in laboratory-based experiments. For this reason, some researchers recently attempted to identify the risk factors for ACL injuries using videos of injured players (Krosshaug et al., 2007; Boden et al., 2009; Hewett et al., 2009; Koga et al., 2010; Koga et al., 2011; Sheehan et al., 2012; Bere et al., 2013).

Compared with uninjured players, athletes who experienced non-contact ACL tears had common features in the sagittal plane. First, the body’s center of mass (COM) was located posterior to the base of support (BOS) at the time of injury (Boden et al., 2009; Sheehan et al., 2012). One study reported that in athletes who experienced ACL injuries, the COM was more than 2 feet posterior to the BOS at the time of landing (Boden et al., 2009). Sheehan et al. (2012) also reported that posterior positioning of the body’s COM above a certain level increases the risk of an ACL injury. They proposed that consistent landing with the COM far posterior to the BOS may act as a screening marker in players at risk for an ACL injury. Second, the trunk and lower limb kinematics in the sagittal plane during landing or stopping events also differed between injured and uninjured athletes. In injured athletes, the trunk angle was more extended, and the hip (measured as the angle between the trunk and the thigh) and limb angles (measured as the angle between the trunk and the vertical line) were more flexed at the initial foot contact (Boden et al., 2009; Sheehan et al., 2012). Moreover, the knee angle was significantly more extended in the injured athletes when the foot was completely flat (Boden et al., 2009). The positions of the trunk, limb, and knee cause the COM to reach far posterior to the BOS, and these findings have been incorporated into ACL injury programs. However, the relationships between the COM position and these kinematic variables, especially during on-field soccer movement, are unknown. Nevertheless, Faunø and Wulff Jakobsen (2006) reported that landing after heading and changing direction after decelerating were particularly associated with soccer-related ACL injury. Accordingly, by observing players’ performance actions that are associated with ACL injuries, we can attempt to identify factors, such as malalignment of the COM relative to the BOS, that may increase the risk of landing or stopping events and lead to such an injury.

The purpose of this study was to determine relationships between the distance from the body’s COM to the BOS and the trunk, hip, and knee kinematics in the sagittal plane in women during soccer. We suggested the following two hypotheses: (1) the COM position correlated with the kinematics at the initial foot contact; and (2) a prediction model of the distance from the COM to the BOS can be created by combining kinematic variables, but coefficient values will differ between each variable.

## Material and Methods

### Data collection

**Figure 1:**
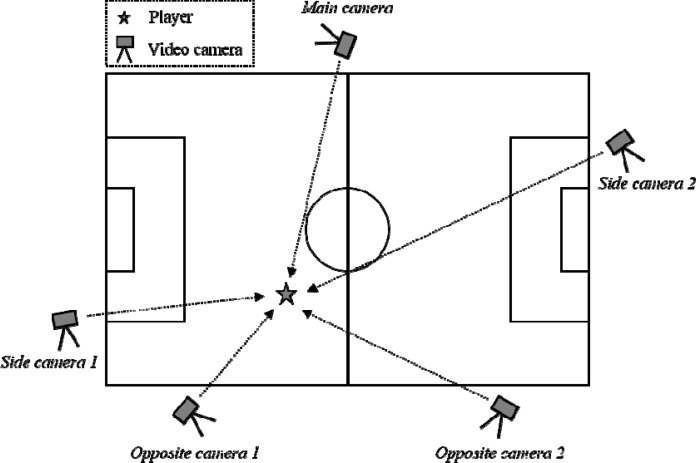
Diagram of the recording procedure in which five digital video cameras were used. Each camera is located around the soccer field at the players’ level

We recorded five female soccer games using ≥3 digital video cameras (HDR-CX590V; Sony, Tokyo, Japan) with a frame rate of 60 Hz. Recordings were made of all players who participated in the games, and 30 players (age 19.0 ± 3.1 years; body height 1.57 ± 0.06 m; body mass 49.36 ± 8.74 kg) were selected for the data analysis. All participants and, if necessary, their parents received an explanation of all the experimental procedures, and written informed consent was obtained. The ethics committee of the Waseda University approved this study. All the players were well trained and belonged to the Division 1 of the Kanto Ladies Soccer League, which is the top woman’s amateur league in East Japan. The cameras were located around the soccer field (approximately 3–10 m from the court line) at the players’ level (approximate height, 1.3 m) to capture their movements from various angles (Figure 1). We recorded the path to the ball and the players around the ball for an entire game using the camera’s pan and tilt feature. All the videos were stored in AVCHD format in high definition (1080i).

Two distinguishing events associated with ACL injuries in soccer players (Faunø and Wulff, 2006) were selected for analysis: (1) a single-leg landing after heading and a skirmish in mid-air; and (2) single-leg stopping from a run after a defensive approach. Three experts in the fields of soccer biomechanics and sports medicine formed the analysis team. The analysis was organized into two stages to identify the events for analysis. First, a researcher identified the aforementioned two events using the main camera (in the center of the field) in 2× slow motion. One hundred twelve events (46 single-leg landing and 66 single-leg stopping events) were selected for further analysis. Forty-one of 112 events involved the dominant (kicking) leg, while the others involved the non-dominant leg. Second, the remaining researchers reviewed all the selected events from the first stage. Video clips from the other angles (i.e., the opposite and side cameras that were not screened in stage 1) were also used to assess whether events should be included in the analysis. The inclusion criteria for the events were as follows: (1) good quality images with the camera angle approximating the sagittal view of the athlete; (2) good visibility of the foot contacting the ground; and (3) an unobscured view of the athletes. Distinct safe landing and stopping events (e.g., landing without a skirmish and double-leg stopping from a run) were excluded from the analysis. Finally, 60 events (29 single-leg landing and 31 single-leg stopping events) met the study criteria. Twenty-four of 60 events involved the dominant leg, while the others involved the non-dominant leg.

### Video editing and analysis

The video recordings were edited using Edius Neo, version 3.5 (Grass Valley LLC, Montreal, Quebec, Canada). For each event, the frame in which the foot initially contacted the ground (initial contact) was captured and stored as a TIFF file for analysis. Image J (National Institutes of Health, Bethesda, MD, USA) was used for all the analyses. For consistency, all measurements were performed by the same researcher.

**Figure 2:**
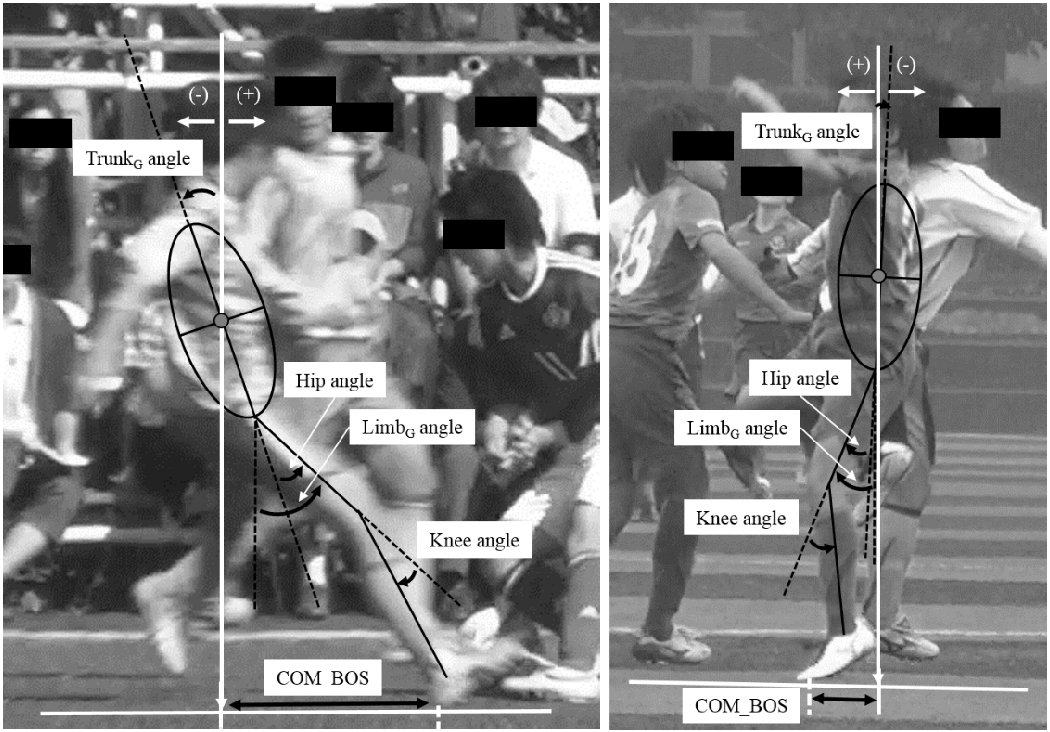
Analysis of the single-leg stopping from a run after a defensive approach (left panel) and a single-leg landing after a heading play with a skirmish mid-air (right panel). COM_BOS, the distance from the center of mass to the base of support

For the sagittal view video analysis, the distance from the COM to the BOS (COM_BOS) and the players’ kinematics at the point of initial contact were measured in reference to that described in previous studies (Boden et al., 2009; Sheehan et al., 2012) (Figure 2). The COM (approximately located at the center of the trunk) was defined as the center of an ellipse delineating the athlete’s trunk, and the BOS was defined as the point bisecting the line of contact between the shoe and the floor at initial contact. The centerline of the trunk was considered the major axis of an ellipse for the COM (represented as the line from the center of the hip joint to the center of the shoulder joint), and the minor axis ran from the anterior aspect to the posterior aspect of the trunk. The COM_BOS (in pixels) was measured along the anterior/posterior direction and normalized by the femur length (represented as the line from the center of the knee joint to the center of the hip joint), because a conversion from pixels to millimeters was not available. We also measured the trunk_G_, limb_G_, and knee joint angles. The trunk_G_ angle was defined as the angle from the vertical line to the centerline of the trunk. The limb_G_ angle was defined as the angle between the vertical line and the centerline of the thigh (represented as the line from the center of the knee joint to the center of the hip joint). The knee angle was defined as the angle between the centerline of the thigh and fibula. A positive trunk_G_ and/or limb_G_ angle indicated that the trunk and/or limb were rotated anteriorly relative to the vertical line. A more detailed description of the method used in this study has been reported previously (Boden et al., 2009; Sheehan et al., 2012).

### Statistical Analysis

All of the descriptive data are expressed as mean ± standard deviation and range. The relationships among the measured variables were determined using the Pearson’s product-moment correlation coefficient. To explore the relationships between the COM position and kinematic variables, stepwise multiple linear regression models were used with the cross-sectional data. The prediction variables entered into the COM_BOS were the trunk_G_, limb_G_, and knee angles. The *F* probability for variable entry was set at .05 and that for variable removal was set at .10. All the statistical procedures were performed using PASW Statistics, version 18.0 for Windows (SPSS, Inc., Chicago, IL, USA), and the statistical significance of all the tests was set at *p* < .05. The statistical power of the tests was stated 0.8 for the differences in the variables noted in this study. In this pilot study, the intraclass correlation coefficients (ICC) were calculated to assess the reproducibility of the measurements for each video frame sequence at two different times. A single rater repeated the measurements for 33 events (12 single-leg landing after heading and 21 single-leg stopping from a run). For the COM_BOS and the trunk_G_, hip_G_, and knee angles, the ICCs were 0.993 (95% confidence interval [CI]: 0.986–0.996), 0.987(95% CI: 0.975–0.994), 0.995 (95% CI: 0.990–0.997), and 0.969 (95% CI: 0.937–0.984), respectively.

## Results

**Figure 3:**
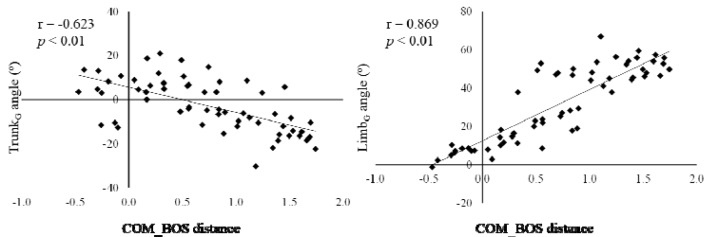
Relationships between the distance from the center of mass to the base of support (COM_BOS) and the trunk (left panel) and limb angles (right panel) at the initial foot contact

Table 1 presents the descriptive characteristics of the COM_BOS and the kinematic variables at the initial foot contact. Table 2 displays the Pearson product-moment correlation coefficients among the variables. The COM_BOS displayed a moderate correlation with the trunk**_G_** angle (r = −0.623, p < .0001, r^2^ = 0.388) and a strong relationship with the limb**_G_** angle (r = 0.869, p < .0001, r^2^ = 0.755) (Figure 3), whereas the knee angle did not correlate with the COM_BOS (r = −0.065, r^2^ = 0.004). The limb**_G_** angle at the initial foot contact was weakly correlated with the trunk**_G_** (r = −0.439, p = .0005, r^2^ = 0.193) and knee angles (r = 0.362, p = .0045, r^2^ = 0.131). The results of the regression analyses are shown in Table 3. The limb**_G_**, knee, and trunk**_G_** angles were the three predictive variables selected in the best regression model (adjusted r^2^ = 0.953, p < .0001, f^2^ = 20.277). The obtained regression equation was as follows: COM_BOS = 0.030 × limb**_G_** angle − 0.023 × knee angle − 0.011 × trunk**_G_** angle + 0.319.

## Discussion

In this study, we described relationships between the distance from the body’s COM to the BOS and kinematic variables during single-leg landing and stopping using video analysis. Our cross-sectional data that were directly obtained from actual game situations, were crucial for understanding on-field sports performance and preventing at-risk movement in women during soccer.

Our first hypothesis was that the COM position correlated with the kinematics at the initial foot contact. Our findings support the relationships between the COM_BOS and two of three variables examined. The COM_BOS displayed a strong and positive correlation with the limb**_G_** angle (r = 0.869, r^2^ = 0.755) and a moderate and negative correlation with the trunk**_G_** angle (r = −0.623, r^2^ = 0.388). Landing on a single leg with the COM far posterior to the BOS on initial ground contact has previously been reported as a risk factor for injury (Sheehan et al., 2012). Therefore, the findings in this research suggest controlling of the trunk and hip positions at the initial contact makes the distance from the COM to the BOS closer that is important for safe landing and stopping.

The mean limb**_G_** angle in the present study (31.90 ± 19.62°) was similar to that recorded in uninjured participants (36 ± 18°) in a previous study by Sheehan et al. (2012). Athletes with ACL injuries tend to have a more flexed hip posture than those without ACL injuries as controls (Boden et al., 2009), especially among women (Krosshaug et al., 2007). Hip muscles assist in the absorption of forces of reaction to the weight of the upper body, while the knee, ankle, and foot absorb ground reaction forces (Boden et al., 2010). Excessive hip flexure at the initial contact with the ground may prevent force absorption, because the angle of the hip joint cannot change the following contact. Additionally, a study on uninjured athletes found that a large limb**_G_** angle makes the tibia slope more vertical relative to the femur (Boden et al., 2009), and this increase in the slope of the posterior tibia plateau may promote anterior tibia shift, which is considered a risk factor for ACL injury. Sheehan et al. (2012) also presented the relationship between the COM_BOS and the limb**_G_** angle in a discriminant analysis, and their findings were similar to those of the current research.

In the present study, the mean trunk**_G_** angle (−2.45 ± 11.75°) was smaller than that recorded in uninjured athletes in a previous study (15 ± 13°); however, it was similar to that recorded in injured athletes (4 ± 14°) (Sheehan et al., 2012). This indicates that landing and stopping events recorded in the present study may have been more dangerous than those recorded in uninjured athletes by Sheehan et al. (2012). A more forward-leaning trunk is more likely to encourage hip and knee flexion (Kobayashi et al., 2006; Shimokochi et al., 2009). Blackburn and Pauda (2008) suggested that active trunk flexion produces a concomitant increase in peak knee and hip angles, and Nagano et al. (2011) reported that more women than men had a smaller trunk inclination angle during a shuttle run cutting task. In a situation where the trunk is upright or leaning backward at the time of ground contact, the ensuing increase in ground reaction force will likely encourage more knee flexion than hip flexion (Hashemi et al., 2011). Interestingly, the risk factors for ACL injury in the present study were found to be linked to the COM position, which is similar to that identified in previous research. However, knee angle was not correlated with COM_BOS (r = −0.065, r^2^ = 0.004), which indicates that a variety of knee flexion patterns may occur during on-field sports. If the trunk and hip positions in the sagittal plane at the initial contact can be controlled during on-field movement, the COM can consistently be positioned near the BOS. An increasing trunk**_G_** angle and a decreasing limb**_G_** angle at initial contact may be important for safe landing and stopping in female soccer players.

In a prospective epidemiologic study by Zazaulak et al. (2007), athletes with a decreased core proprioception or neuromuscular control of the trunk were found to be at an increased risk of knee injury. This indicates that compromised function of the trunk and hip stabilizers may underlie the ACL injury risk in female athletes (Myer et al., 2008). Myer et al. (2008) developed a neuromuscular training protocol consisting of five exercise phases to target deficits in trunk and hip control. Such targeted neuromuscular training may be useful for developing control not only of the limb and trunk positions but also of the COM_BOS during dynamic activities.

Our second hypothesis was supported by the stepwise multiple regression analysis for predicting the COM_BOS using kinematic variables. The first regression analysis revealed that 95.3% of the variance in the COM_BOS was explained by the limb**_G_**, knee, and trunk**_G_** angles (adjusted r^2^ = 0.953, f^2^ = 20.277). Based on the standardized coefficient values, the limb**_G_** angle (standardized coefficient = 0.919) had a greater influence on the COM_BOS than the knee (standardized coefficient = −0.393) and the trunk**_G_** angles (standardized coefficient = −0.211). This indicates that the limb position is particularly important in determining the COM position. In addition, these data suggest that the COM_BOS represents a comprehensive assessment tool that reflects the three kinematic variables. Although the kinematic variables (i.e., trunk, limb, and knee angles) were considered risk factors for ACL injury, it is time consuming to analyze all three different parameters separately. The COM_BOS was recommended as a screening tool for athletes at risk for ACL injury in previous research (Sheehan et al., 2012). This current study also supports the use of COM_BOS, because an easy-to-use tool is preferred during on-field assessments.

Some limitations must be considered when interpreting the findings of the present study. First, it is not easy to identify anatomical landmarks without markers in players who are clothed; however, the reliability measures were excellent in this pilot study. Although the accuracy of each current variable was somewhat lower than that with a three-dimensional biomechanical analysis in the laboratory setting, our data, which were directly obtained from real game situations, were crucial for understanding on-field sports activities. Significant information related to performance can be obtained or observed in the actual games that cannot be simulated in experimental environments. Therefore, analyzing the players’ kinematics during competition or games quantitatively can clarify how the players should perform during complex and dynamic activities. Another limitation is that, there was some variation in camera angles, and consequently, the cameras may not have perfectly captured the players in the sagittal plane. Previous studies that used video analysis discussed the same difficulties (Boden et al., 2009; Hewett et al., 2009). However, even in a worst-case scenario in which the participants are 30° away from the sagittal plane, a significant difference in the COM_BOS may not occur (Sheehan et al., 2012). In this study, an analysis team consisting of three experts was formed to ensure a careful selection of the criteria. We believe that video analysis using a computerized technique is innovative for field-based studies, because visual inspection approaches are inaccurate (Krosshaug et al., 2007). Finally, it was not possible to determine the exact moment of initial foot contact, because of a relatively low frame rate (60 Hz) used in this study. Millisecond differences exist in the captured sequences of initial contact. Although two-dimensional video analysis included the aforementioned limitations, we can reconstruct current motions using a sophisticated three-dimensional video analysis technique (Krosshaug et al., 2005). If further analysis is necessary to obtain highly accurate kinematics, synchronized videos captured from various angles will provide more detailed data during on-field soccer movements of female players.

## Conclusions

This study reported on relationships between the distances from the body’s COM to the BOS and kinematics in the sagittal plane during soccer games in women. The COM_BOS displayed a moderate and negative correlation with the trunk angle (r = −0.623, p < .0001, r^2^ = 0.388) and a strong and positive correlation with the limb angle (r = 0.869, p < .0001, r^2^ = 0.755). In addition, the first regression analysis revealed that 95.3% of the variance in the COM_BOS can be explained by the limb, knee, and trunk angles (adjusted r^2^ = 0.953, p < .0001, f^2^ = 20.277). The COM_BOS is a comprehensive assessment tool that reflects a combination of the three kinematic variables. These findings suggest that an increased trunk angle and a decreased limb angle at initial contact are associated with a safe COM position during single-leg landing and stopping. Neuromuscular training focused on trunk and hip stabilizers may be useful for controlling the trunk and lower limb positions during dynamic activities.

## Figures and Tables

**Table 1:** COM_BOS distance and the trunk, limb, and knee angles at the initial foot contact

Variables	Mean ± SD	Range
COM_BOS/femur	0.72 ± 0.64	−0.47 to 1.74
Trunk_G_ angle, º	−2.45 ± 11.75	−30.11 to 21.04
Limb_G_ angle, º	31.90 ± 19.62	−1.20 to 66.99
Knee angle, º	25.49 ± 11.03	4.45 to 53.21

COM_BOS, the distance from the center of mass to the base of support.

The value COM_BOS/femur length is a scaled value and as such, is unitless, as indicated by Sheehan et al. (2012). SD, standard deviation

**Table 2:** Correlation coefficients among the variables

	COM_BOS	Trunk_G_ angle	Limb_G_ angle	Knee angle
COM_BOS	1.000			
Trunk_G_ angle	−0.623^**^	1.000		
Limb_G_ angle	0.869^**^	−0.439^**^	1.000	
Knee angle	−0.065	0.023	0.362^**^	1.000

** *p* < 0.01

COM_BOS, the distance from the center of mass to the base of support

**Table 3:** Stepwise linear regression analysis model for the COM_BOS distance

COM_BOS distance [Adjusted r^2^ = 0.953]				

Predictor	Coefficient	Standardized coefficient	*p* value	VIF
Intercept	0.319			
Limb_G_ angle	0.030	0.919	<0.001	1.494
Knee angle	−0.023	−0.393	<0.001	1.208
Trunk_G_ angle	−0.011	−0.211	<0.001	1.299

COM_BOS, the distance from the center of mass to the base of support;

VIF, variance inflation factor
